# Rates of homology directed repair of CRISPR-Cas9 induced double strand breaks are lower in naïve compared to primed human pluripotent stem cells

**DOI:** 10.1016/j.scr.2020.101852

**Published:** 2020-07

**Authors:** Benjamin T. Dodsworth, Klas Hatje, Claas Aiko Meyer, Rowan Flynn, Sally A. Cowley

**Affiliations:** a Sir William Dunn School of Pathology, University of Oxford, South Parks Road, OX1 3RE, United Kingdom; b Roche Pharma Research and Early Development, Roche Innovation Center Basel, Basel, Switzerland; c Censo Biotechnologies, Roslin Innovation Centre Charnock Bradley Building, Easter Bush Campus EH25 9RG, United Kingdom

**Keywords:** Human, Naïve, Ground state, Pluripotent, Stem cell, Pluripotency, Gene editing efficiency, Homologous recombination, Homology directed repair, Non-homologous end joining, HDR, HR, NHEJ

## Abstract

Gene editing in human pluripotent stem cells (hPSC) is a powerful tool for understanding biology, for drug discovery and gene therapy. Naïve hPSC have been suggested to be superior for gene editing compared to conventional ‘primed’ hPSC. Using droplet digital PCR, we uncover the kinetics of Cas9-induced double strand break repair in conventional hPSC. Cut but unrepaired alleles reach their maximum after 12–24 h. Homology directed repair plateaus after 24 h, whereas repair by non-homologous end joining continues until 48 h after Cas9 introduction. Using this method, we demonstrate that the rate of homology directed repair to resolve Cas9-induced double strand breaks is 40% lower in naïve hPSC compared to conventional hPSC, correlating with, and feasibly explained by, a higher number of cells in G_1_ phase of the cell cycle in naïve hPSC. Therefore, naïve hPSC are less efficient for CRISPR/Cas9-mediated homology directed repair.

## Highlights

• Kinetics of Cas9-induced double strand break repair in conventional hPSC.
• Homology directed repair to resolve Cas9-induced double strand breaks is 40% lower in naïve hPSC compared to conventional hPSC.
• Naïve hPSC (4iLA) have a higher number of cells in G_1_ phase of the cell cycle.

## Graphical abstract

**Figure:**
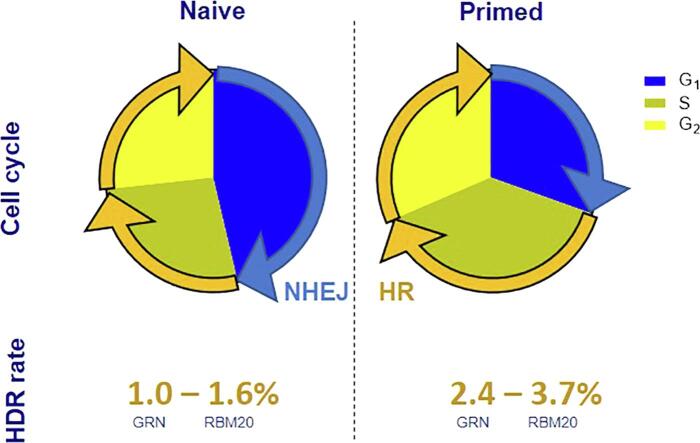

## 1 Introduction

Naïve mouse pluripotent stem cells (PSC) can be more readily genetically manipulated by homologous recombination (HR) than primed mouse epiblast stem cells (EpiSC) or conventional, primed human PSC (Zwaka and Thomson, 2003). Therefore, the question has been raised whether the recently described human naïve pluripotent stem cells can also be more readily genetically manipulated. Studies that have addressed this question to date do not directly explore whether the rates of HR are different between human naïve and primed hPSC (Gafni et al., 2013, Buecker et al., 2010).

Miyaoka *et al.* developed a droplet digital PCR assay which accurately measures whether a nuclease induced double strand break (DSB) is repaired using a template strand via homology directed repair (HDR) or with an indel formation caused by canonical non-homologous end joining (C-NHEJ; or by microhomology-mediated end joining) (Miyaoka et al., 2016). This assay was designed for use with TALENs and plasmid-based Cas9 delivery, but since that publication, higher gene editing efficiencies have been made possible using Cas9 protein in place of plasmids (Jacobi et al., 2017).

Here, we adapt Miyaoka *et al*.’s assay (Miyaoka et al., 2016) to work with ribonucleoprotein complexes (RNPs, Cas9 protein complexed with gRNA and tracrRNA) and uncover that naïve hPSC can be efficiently modified by genome editing but have lower rates of single stranded oligodeoxynucleotide (ssODN)-mediated HDR than primed hPSC, likely in part due to fewer cells in S/G_2_ phase of the cell cycle.

## 2 Materials and methods

Reagents were from ThermoFisher unless stated otherwise. For more detail see Supplementary Experimental Procedures.

### 2.1 Cell culture

hPSC from 4 donors were derived and cultured as previously published (Haenseler et al., 2017, Jonikas et al., 2018, Fernandes et al., 2016) (Table S1). Briefly, cells were cultured at 7% CO_2_ and 5% O_2_ with daily media changes. Naïve cells were grown on irradiated (30 gy) CF1 mouse embryonic fibroblasts (MEFs) (Millipore; PMEF-CFL) as previously described (Theunissen et al., 2014) with minor adaptations from subsequent publications: IM-12 from the original Theunissen *et al.* was omitted in 4iLA (Theunissen et al., 2016); 70% media changes were implemented (Collier et al., 2017). Primed cells were grown on geltrex (A1413302) in E8 (A15169-01).

### 2.2 Assay to measure NHEJ and HDR

The assay to measure ssODN-mediated homology directed repair (HDR)) and non-homologous end joining was based on Miyaoka et al. (Miyaoka et al., 2016), adapted here to work with Alt-R® CRISPR-Cas9 System procured from Integrated DNA Technologies and applied to hPSC.

### 2.3 Droplet digital PCR

Droplet digital PCR was performed as in Miyaoka *et al.* (Miyaoka et al., 2016). To measure cut but not repaired alleles, the NHEJ, HDR and dark probes were omitted and the TaqMan™ Copy Number Reference Assay (RNase P; 4403326) added to the reaction mix. The ratio of the two reference probes informs how many alleles are missing.

### 2.4 PI staining

Propidium Iodide (PI) staining for cell cycle analysis followed Krishan’s protocol with minor modifications (see supplementary methods) (Krishan, 1975).

### 2.5 Results and discussion

**Fig. 1:**
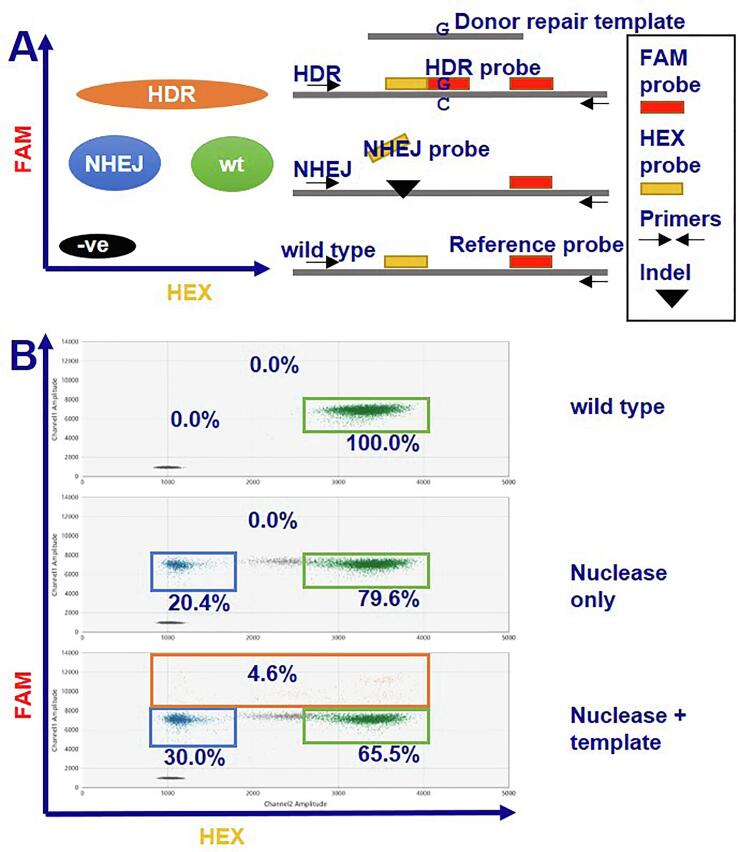
Measuring homology directed repair (HDR) and non-homologous end joining (NHEJ) of a Cas9 induced double strand break by droplet digital PCR. Method adapted from Miyaoka et al. (Miyaoka et al., 2016). (A) The reference probe distinguishes droplets containing the amplicon from negative droplets. Unedited amplicons allow binding of both the NHEJ and reference probe and are the double positive FAM + HEX population. Indel formation due to NHEJ prevents the NHEJ probe from binding, resulting in the single positive FAM population. When the template strand is used for repair, a single nucleotide difference is introduced. This allows binding of the HDR probe and leads to the FAM++ population. Exact design of the probes are previously published (Miyaoka et al., 2016). (B) Illustrative data (primed SFC856-03–04, locus *GRN*, guide F1) showing the gating used to distinguish different populations. The upper plot shows unedited wild type cells lead to a FAM + HEX population. Adding the nuclease (and guide) causes cutting and repair by NHEJ. The subsequent emergence of erroneously NHEJ repaired DNA leads to an additional population of single positive FAM droplets (middle plot). Only if the template oligo and nuclease are introduced the HDR FAM++ population appears (lower plot). Uptake of the RNP is often improved when an oligo is present, explaining the increase in the NHEJ population.

To measure ssODN-mediated HDR and NHEJ, we used a droplet digital PCR based assay (Fig. 1). Miyaoka *et al.* designed the assay with plasmid based (pX330) Cas9. Here, we used HiFi Cas9 protein, which allows very accurate measurement of repair over time. Further, by measuring the relative copy number of our locus compared to the control RNaseP locus, we could calculate the fraction of cut but not repaired alleles.

**Fig. 2:**
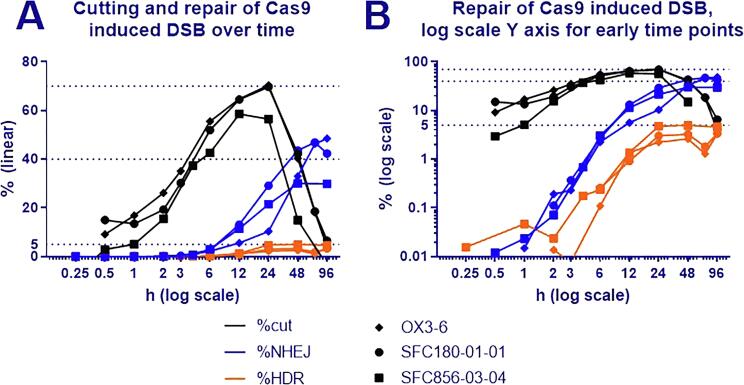
**Repair of Cas9-induced double strand break over time.** Cas9 protein, guide RNA against the GRN locus and a template oligo were transfected into three conventional (primed) iPSC lines by electroporation. The emergence of cut but not repaired DNA is measured by comparing copy number of the GRN locus to the control RNaseP locus. Erroneous repair is detected by loss of probe binding and is marked %NHEJ. Repair by homologous recombination using the template strand causes an extra probe binding and is reported as %HDR. Missing datapoints indicate values ≤ 0. (A) The data is expressed with a linear Y axis (B) as well as with a log scale Y axis to show the earlier time points more clearly.

Primed hPSC from 3 donors were electroporated with HiFi Cas9, guide RNAs and repair template oligo for the GRN locus, in order to establish when repair first occurs post electroporation and for how long new repair events continue to occur. Pellets of electroporated cells were recovered over a 96 h time course, DNA was extracted, and the type and extent of repair determined by droplet digital PCR (Fig. 2A). Over the 96 h time course, the %cut rose sharply within the first 12 h and began to decrease after 24 h. After 96 h, no cut but unrepaired DNA remained. Repair with indel formation occurred at low levels as early as 30 min after transfection, reaching 1% after 4 h and plateauing at 30% after 48 h (Fig. 2B). Measurement of repair using the template strand seems variable at very low levels, but a clear increase is seen at 4 h and beyond. After 24 h, HDR levels plateau at 5%. Note that the overall level of template-mediated repair is relatively low using ssODN, while other systems for template delivery can yield higher rates of gene targeting (Martin et al., 2019), so our results do not necessarily extend to other modes of gene targeting, which are mechanistically distinct. To the best of our knowledge, this is the first dataset investigating Cas9 kinetics in hPSC and fits well with previously published Cas9 kinetics results on the human myeloid leukaemia cell line K562 (Brinkman et al., 2018).

**Fig. 3:**
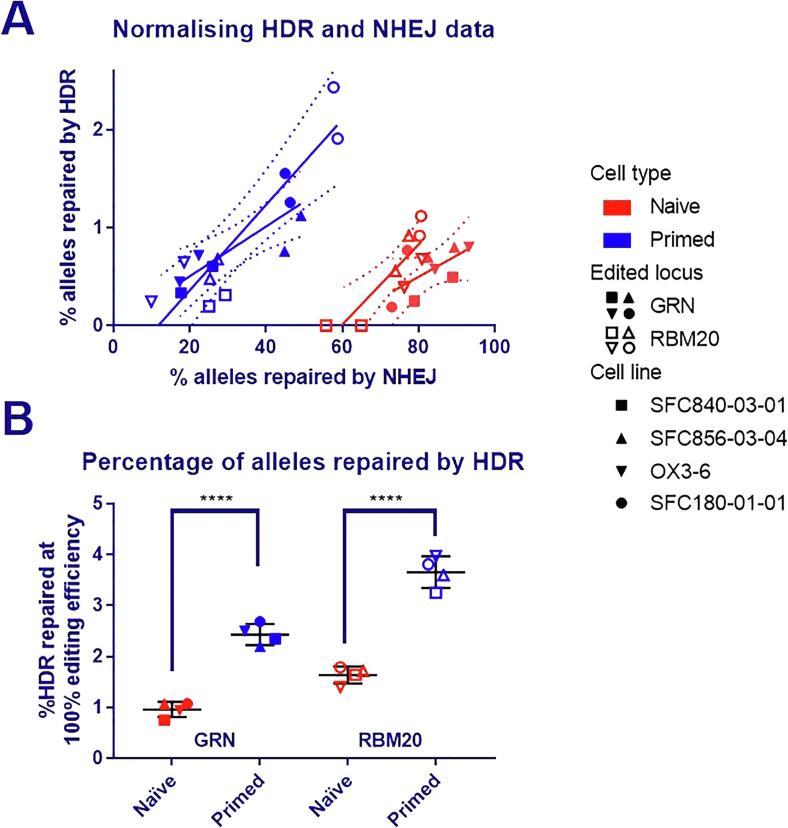
**Comparison of DNA repair in naïve and primed hPSC. To remove variability (e.g. from different RNP uptake efficiency), the rate of HDR was normalised to the rate of NHEJ repair.** (A) In order to normalise, the relationship between %HDR and %NHEJ repair was established. The %HDR and %NHEJ repaired alleles for each electroporation were plotted and a line was fitted by linear regression for each locus and for naïve and primed cells. For each locus, the pooled slope for naïve and primed electroporations was calculated (RBM20 = 0.043 and GRN = 0.025). (B) The pooled slope was used to calculate the normalised HDR rate (= %HDR - (%NHEJ × slope)) and the result scaled to show the maximum possible editing efficiency (%HDR+%NHEJ = 100) and plotted. Each symbol is the mean of 2 electroporations of the same cell line and error bars show the standard deviation of the 4 biological replicates. A two-tailed students *t*-test informed significance (**** P ≤ 0.0001). The underlying data before normalisation is shown in Supplementary Fig. S1.

We then used this assay to compare repair of DSBs in naïve and primed hPSC. Since we were focussing on two very different cell types, we compared the proportion of alleles repaired by ssODN-mediated HDR to mutagenic NHEJ, thus removing variation introduced by, for example, altered Cas9 uptake efficiency (Fig. 3A). Naïve cells established using the Theunissen 4iLA protocol were used in this experiment (Theunissen et al., 2014, Theunissen et al., 2016). Naïve and their corresponding primed cell lines were transfected with Cas9, guide RNAs and template strands for either GRN or RBM20. Each transfection was performed in duplicate and an extra non-specific template control using an unspecific oligo (“electroporation enhancer”) instead of the template was included. After 4 days, DNA was extracted from the cells and HDR and NHEJ repair was measured using the droplet digital PCR assay. For both loci, the proportion of ssODN-mediated HDR repaired alleles were significantly lower (40% lower, p < 0.0001, two-tailed students *t*-test) in naïve cells (Fig. 3B). Experiments were performed with 3 karyotypically normal lines, as well as naïve converted SFC856-03-04, which contained a duplication in the small arm of chromosome 12.

**Fig. 4:**
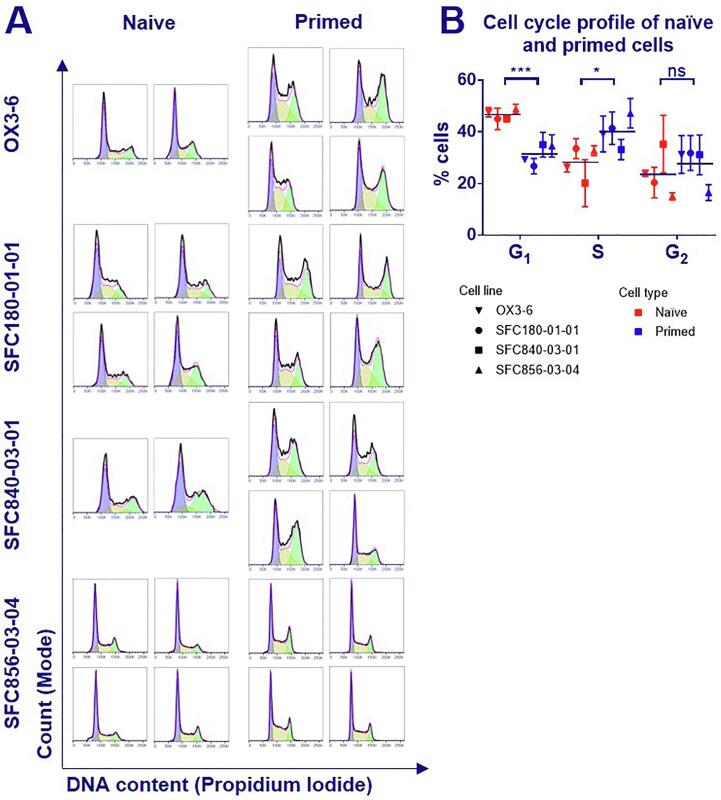
**Cell cycle analysis of naïve and primed hPSC.** The cells in naïve and primed states were fixed (2–4 separate replicates, fixed on different days) and stained with propidium iodide (PI) to analyze the proportion of cells in each stage of the cell cycle. (A) Histograms of the DNA content are plotted with the measured DNA content displayed as the black lines. The Dean-Jett-Fox method was used to calculate proportions of cells in each stage of the cell cycle. In this mathematical model, cells predicted to be in G_1_ are marked in purple, yellow are in S phase and green in G_2_. The pink line shows the sum of these populations. (B) The percentage of cells in G_1_, S and G_2_ phase are plotted for each cell line. Symbols represent the mean of 2–4 replicates with error bars showing the standard deviation. The overall mean of all 4 cell lines is displayed as a line. Significance informed by a two-tailed students ratio paired *t*-test on 4 biological replicates (ns = p > 0.05; * = p ≤ 0.05; *** = p ≤ 0.001).

Since rates of HDR are cell cycle dependent (Rothkamm et al., 2003, Kass and Jasin, 2010) we next explored cell cycle dynamics. Naïve and primed cells were fixed (each replicate on a different day), stained with propidium iodide (PI) and their DNA content measured by flow cytometry. Cell debris and duplets were removed by gating and the Dean-Jett-Fox method was used to calculate proportions of cells in different stages of the cell cycle (Fig. 4 A,B) (Fox, 1980). The proportion of cells in G_1_ phase was 1.4-fold higher in naïve cells. Since HDR occurs mostly in late S and G_2_ phases, changes in cell cycle dynamics affects rates of HDR (Rothkamm et al., 2003, Kass and Jasin, 2010, Shao et al., 2012, Sun et al., 2012, Huertas and Jackson, 2009, Falck et al., 2012). Whilst we cannot determine here whether the difference in cell cycle is an inherent property of these cell states, or whether it is a consequence of the different media used for each state, the difference in cell cycle is likely to be at least a contributor to the lower rate of HDR observed.

## 3 Conclusion

We have demonstrated high rates of Cas9-induced targeted double strand breaks in hPSC and shown their repair over time. Our results concur with current estimates of Cas9 efficiency and represent a useful guide for setting up experiments in hPSC.

Prior publications imply that naïve hPSC may be superior for gene editing. We uncover that rates of ssODN-mediated HDR are lower in naïve hPSC, feasibly due to more cells in G_1_ phase of the cell cycle. Naïve hPSC are therefore less efficient for HDR-based gene editing such as gene correction.

## 4 Funding

BBSRC grant number BB/L015447/1; EU IMI STEMBANCC grant number 115439; The Wellcome Trust WTISSF121302; Oxford Martin School LC0910-004; MRC Dementias Platform UK Stem Cell Network Capital Equipment MC_EX_MR/N50192X/1; Innovative Medicines Initiative Joint Undertaking 115,439 (FP7/2007e2013).

## 5 Data Availability

All data generated or analysed during this study are included in this published article (and its Supplementary Information files).

## CRediT authorship contribution statement

**Benjamin T. Dodsworth:** Conceptualization, Methodology, Formal analysis, Writing - original draft. **Klas Hatje:** Formal analysis, Writing - review & editing. **Claas Aiko Meyer:** Conceptualization, Funding acquisition. **Rowan Flynn:** Conceptualization, Writing - review & editing, Funding acquisition. **Sally A. Cowley:** Conceptualization, Writing - review & editing, Project administration, Funding acquisition, Supervision.

## Declaration of interests

The authors declare that they have no known competing financial interests or personal relationships that could have appeared to influence the work reported in this paper.
