# Supplementary material for: Rates of homology directed repair of CRISPR-Cas9 induced double strand breaks are lower in naïve compared to primed human pluripotent stem cells
Source: Stem Cell Res. 2020 Jul;46:101852. doi: 10.1016/j.scr.2020.101852 (PMC7347009; doi:10.1016/j.scr.2020.101852)
Supplement: Supplementary data 1 [file mmc1.pdf]

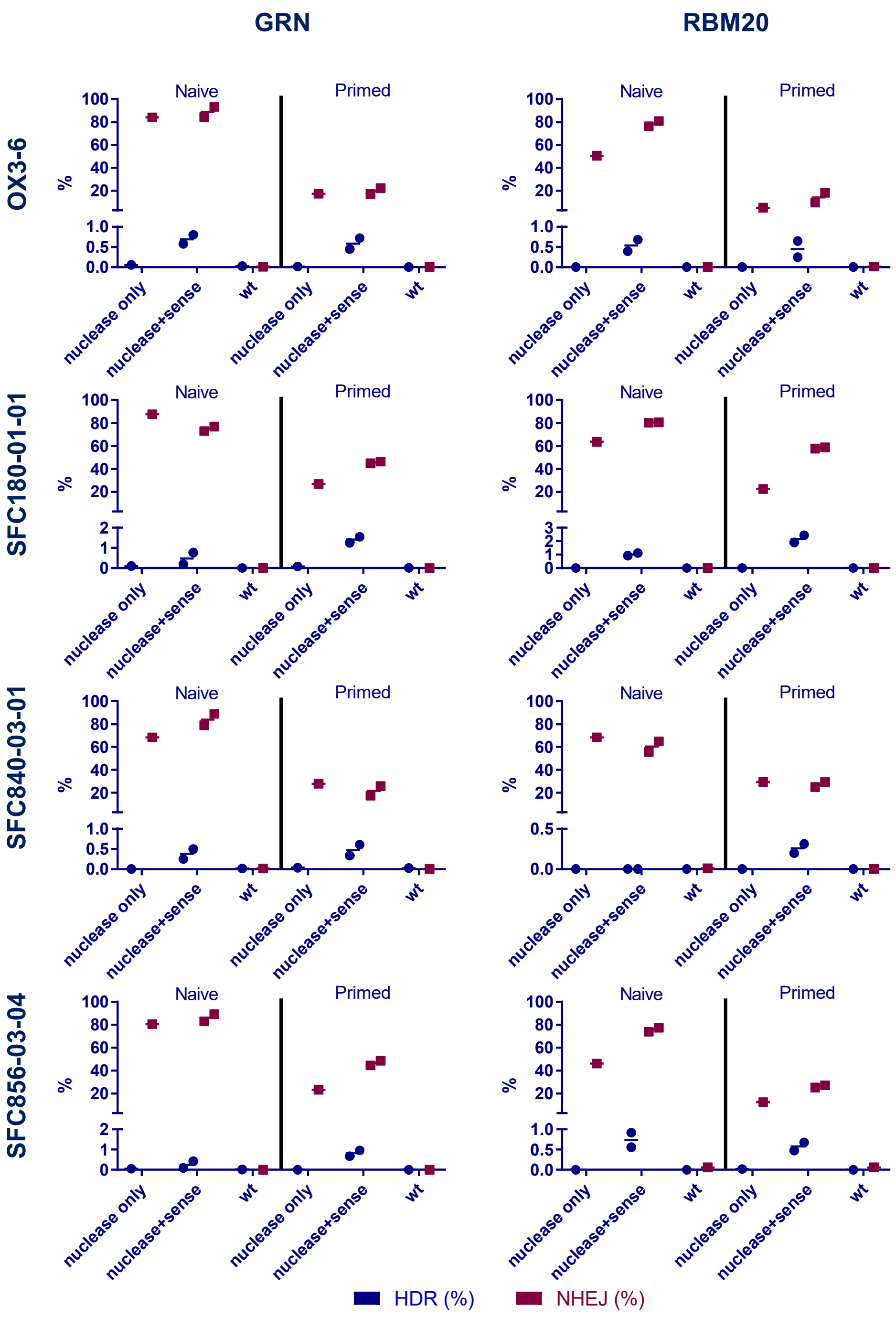

Figure S1: Underlying data to Figure 3. As expected, neither NHEJ nor HDR is observed in the wild type controls and HDR is not observed in the nuclease only control.

line name	alternative name	reprogramming method	Reference
OX3-6		Sendai (Nakanishi)	7
SFC180-01-01		Sendai (CytoTune 2)	6
SFC840-03-01	AH017-11	Sendai (CytoTune 1)	8
SFC856-03-04		Sendai (CytoTune 2)	6

Table S1: Origins of iPSC lines used in this publication.

Supplemental Methods

Assay to measure NHEJ and HDR

The assay requires the introduction of a Cas9 induced double strand break, extraction of genomic DNA and uses a droplet digital PCR based method for HDR and NHEJ quantification.

To make RNPs, Alt-R® CRISPR-Cas9 crRNA and Alt-R® CRISPR-Cas9 tracrRNA (1072532; Integrated DNA Technologies) were resuspended in 100 µM in the supplied duplex buffer. To allow binding of crRNA and tracrRNA, 2 volumes of each were added to 1 volume of duplex buffer. The mixture was heated to 95°C for 5 minutes, removed from heat and allowed to cool on the bench. This guide RNA mixture was used directly or stored at -20°C for future use. Alt-R® S.p. HiFi Cas9 Nuclease (1081060; Integrated DNA Technologies) was diluted by adding 3 volumes to 2 volumes of buffer R (from the Neon electroporation kit). Diluted Cas9 was then mixed in equal volumes with the prepared guide RNAs and allowed to incubate for 20 minutes to make the ribonucleoprotein (RNP). The single stranded template oligo (GRN = CGGCTGGCTACACCTGCAACGTGAAGGCTTGAT CCTGCGAGAAGGAAGTGGTCTCTGCCC, RBM20 = ACAGATATGGCCCAGAAAGGCCGC GGTCTAGTAGTCCGGTGAGCCGGTCACTCTCCCCGA) and the non-targeting oligo control (Alt-R® Cas9 Electroporation Enhancer; 1075915; Integrated DNA Technologies) were resuspended in duplex buffer to 100 µM and stored at -20°C for further use. The oligos were further diluted in buffer R to 10.8 µM and 1 volume of RNP was mixed with 2 volumes of the template oligo or the control Alt-R® Cas9 Electroporation Enhancer. 3 µL (for 10 µL electroporation tip) or 30 µL (100 µL tip) of the mixture was added to a sterile tube ready for electroporation.

Electroporation of cells with RNPs was performed. Cells were lifted by washing with PBS and adding 1 mL Accutase per well of a 6 well plate or 3 mL per 10 cm dish. After 4-5 minutes of incubation, the cells were washed through a 40 µm cell strainer and diluted 1:5 with 10% ES FBS in KO DMEM to inactivate the enzyme. They were then spun at 400 x g for 5 minutes and the supernatant was subsequently discarded. Cells were resuspended in 1 mL PBS (2 mL for the time course experiment), counted (diluted 1:10 for counting), 1 mL transferred into an Eppendorf and spun. After discarding the supernatant, cells were resuspended to 2.2 x 107 cells/mL in buffer R.

For the time course experiment 5 tubes of 30 µL RNP & template were prepared as above, 90 µL primed cells added to each and electroporated using the 100 µL neon electroporation tip (1400V, Pulse width 20, Pulse number 1). After electroporation, cells were immediately pooled into 19.5 mL mTeSR + ROCKi. (The no template control was performed using just one tube and transferred to 8 mL mTeSR + ROCKi.) For early time points (15 min, 30 min, 1h, 2h, 4h, 6h, 12h, 24h), 2 mL (1 x 10^6) electroporated cells were plated onto a well each of a 6 well plate precoated with geltrex (1.1 x 10^5 cells/cm^2). For time points 48h, 72h and 96h, cells were diluted 1:2 before plating in 6 well format on geltrex precoated wells. The wild type and no template controls were plated at 5 x 10^5 cells per well onto geltrex precoated wells (5.5 x 10^4 cells/cm^2). The 15 min time point was not plated but instead placed in a tube ready for pelleting, this time point was omitted in 2/3 replicates. At each other time point, cells were lifted with accutase as previously explained, pelleted by spinning for 1 min at 1000 x g and frozen at -80°C. For early timepoints up to 24h the supernatant was included in the tube, so any floating cells were included in the pellet.

To measure differences in repair pathways between naïve and primed cells, 9 µL cells were added to the 3 µL cas9 RNP + template prepared above and electroporated in a 10 µL electroporation tip. The optimal electroporation programme was used for naïve (1150V, Pulse width 30, Pulse number 2) and primed (1400V, Pulse width 20, Pulse number 1) cells. Cells were immediately transferred into 12 well plates (either pre-treated with geltrex for primed cells or containing MEFs for naïve) containing the appropriate medium with ROCKi. After 4 days, cells were pelleted and DNA extracted using the DNeasy Blood and Tissue kit (69506; Qiagen).

Cell cycle analysis by Propidium Iodide staining

To measure the relative number of cells in each stage of the cell cycle, propidium iodide staining was performed. Each replicate was collected on a different day by lifting the cells with accutase as above and passing the cells through the cell strainer into 10% FBS. Cells were transferred to a 15 mL conical tube, counted, spun and resuspending in 1.5 x 10^6 in PBS. 1 mL was transferred to a new conical tube, spun and the supernatant discarded. Cells were then thoroughly resuspended in cold PBS, ensuring no cell clumps. Cells were fixed by adding 5 mL ice cold (-20°C) 100% ethanol dropwise to the tube while gently vortexing. Fixed cells were kept at -20°C for up to several weeks, until several samples were ready for staining. Staining of the DNA was performed by spinning the cells, removing the supernatant, and resuspending the cells in 100 µL PI staining solution (50 µg/mL PI + 100 µg/mL RNAse A in PBS; 556463; BD Biosciences). Cells were filtered using a 40

μm filter (352235; BD Biosciences) to remove cell clumps and incubated at 4°C in the dark for 20 min. They were then analysed on a flow cytometer (BD LSRFORTESSA X-20; BD Biosciences). (Krishan 1975)
